# Distal Radial Artery Aneurysm of the Dorsal Branch with Ulnar Artery Occlusion: A Case Report

**DOI:** 10.3400/avd.cr.25-00064

**Published:** 2026-02-26

**Authors:** Yoshihiko Onishi, Akihiro Sasahara, Saya Ishikawa, Kenjiro Sakaki, Ko Shibata, Masaki Nie, Kuniyoshi Ohara

**Affiliations:** Department of Cardiovascular Surgery, Ebina General Hospital, Ebina, Kanagawa, Japan

**Keywords:** radial artery aneurysm, ulnar artery occlusion, palmar arterial arch

## Abstract

We report a rare case of a radial artery aneurysm associated with ulnar artery occlusion in an 80-year-old female. Preoperative imaging showed poor visualization of the palmar arch and absent ulnar flow, prompting aneurysmectomy and radial artery reconstruction using a vein graft. Intraoperative findings revealed good retrograde flow, suggesting preserved distal perfusion. This case highlights the importance of accurate preoperative assessment of hand arterial anatomy, particularly the palmar arch, in determining the need for revascularization in radial artery aneurysms with compromised collateral circulation.

## Introduction

Most cases of radial artery aneurysms are iatrogenic pseudoaneurysms typically arising as complications of transradial interventions, while true radial artery aneurysms are exceedingly rare.^1)^ Aneurysms of the upper extremities account for approximately 1% of all aneurysms, with radial artery aneurysms comprising only 2.9% of these cases.^2)^ We report a complicated case of radial artery aneurysm with ulnar artery occlusion, accompanied by a literature review.

## Case Report

An 80-year-old female with a history of percutaneous coronary intervention (PCI) for unstable angina via the right radial artery 4 years prior presented with a gradually enlarging, painless mass over the dorsal aspect of her left thumb, first noted approximately 1 year earlier. She was referred after ultrasonography suggested a radial artery aneurysm.

**Fig. 1:**
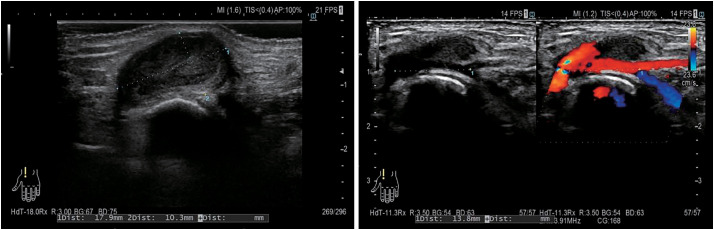
Preoperative ultrasound images. Preoperative ultrasound showing an 18 × 10 mm saccular aneurysm in the radial artery with pulsatile flow.

**Fig. 2:**
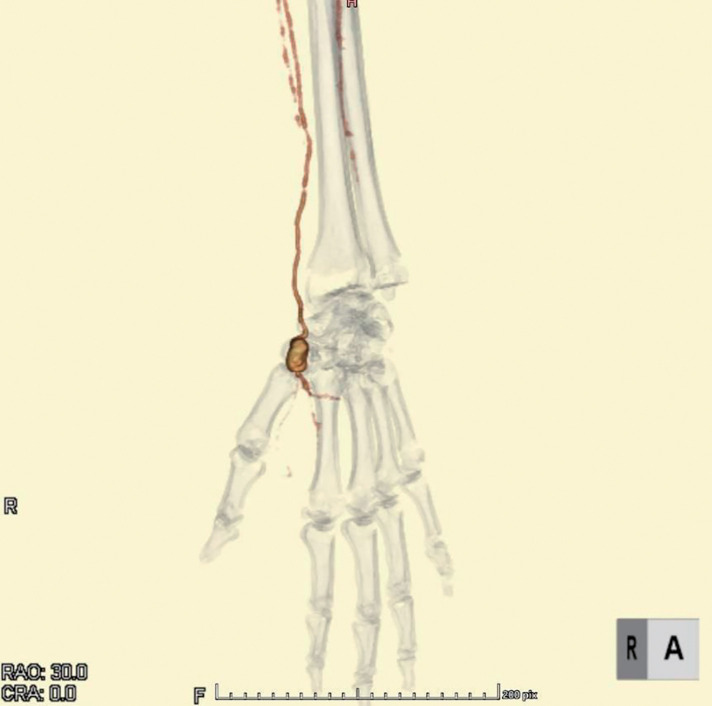
3D image of preoperative contrast-enhanced CT scan. A saccular aneurysm in the left radial artery with ulnar artery occlusion and poor visualization of the palmar arch. 3D: 3-dimensional; CRA: coronary angiography; CT: computed tomography; RAO: retinal artery occlusion

**Fig. 3:**
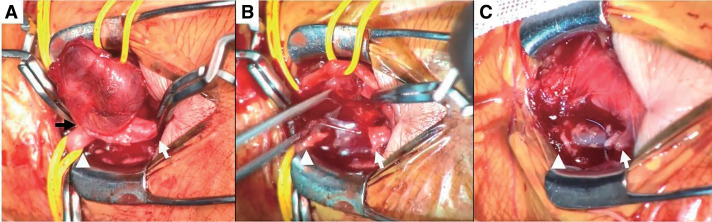
Intraoperative images. White arrowhead: proximal side of the aneurysm; white arrow: distal side of the aneurysm. (**A**) After clamping the aneurysm proximally and distally, the lesion was found to have a stalk approximately 5 mm in diameter (black arrow). (**B**) Following aneurysm resection, good retrograde blood flow from the distal side was observed during proximal occlusion. (**C**) After revascularization using the left radial cephalic vein.

Physical examination revealed a 1.5-cm pulsatile mass at the anatomical snuffbox of the left hand. No neurological deficits or signs of ischemia were observed. The Allen test demonstrated absent ulnar flow on the left. She had no history of trauma, recent infection, or catheterization involving the left upper limb. Her medical history included type 2 diabetes mellitus, hypertension, dyslipidemia, asymptomatic carotid stenosis, atrial fibrillation, and chronic renal failure. Ultrasound revealed an aneurysm measuring 18 × 10 mm, located approximately 14 mm proximal to the distal bifurcation of the left radial artery (**Fig. 1**). The ulnar artery showed no detectable flow signal on Doppler examination. Contrast-enhanced computed tomography (CT) demonstrated a saccular aneurysm arising from the snuffbox and complete occlusion of the left ulnar artery. The palmar arterial arch was poorly visualized (**Fig. 2**), and these findings remained unchanged in the delayed phase. Due to the risk of rupture and potential distal ischemia resulting from compromised collateral flow, we opted for aneurysmectomy with vascular reconstruction. Preoperative catheter angiography was withheld due to concerns regarding the risk of radial artery occlusion associated with the procedure. Under local anesthesia, the aneurysm was resected via a direct dorsal incision. A 5-mm stalk was identified (**Fig. 3A**), and the aneurysm was excised in its entirety. Despite preoperative imaging suggesting inadequate collateral flow, good retrograde flow was observed intraoperatively from the distal side (**Fig. 3B**). Given the preoperative concern for insufficient perfusion, we opted for arterial reconstruction. Due to the long distance between the arterial stumps, an interposition graft using the left cephalic vein was performed, which was harvested through the same operative exposure (**Fig. 3C**). During intraoperative clamping and after completing the reconstruction, left finger oxygen saturation remained at 98%–100%, equivalent to the contralateral side. The patient exhibited no neurological deficits and no pallor. Postoperatively, these findings persisted. She was discharged on postoperative day 2 and has had an uneventful follow-up without recurrence.

## Discussion

True radial artery aneurysms may be idiopathic, atherosclerotic, mycotic, or associated with underlying vascular anomalies. When located distally, they often arise in the anatomical snuffbox.^2)^ Similar to visceral artery aneurysms, peripheral aneurysm formation can occur in response to increased collateral flow necessitated by proximal arterial stenosis or occlusion. In such cases, elevated shear stress may lead to progressive vessel wall remodeling and aneurysmal degeneration.^3)^

The hallmark presentation of radial artery aneurysms is a painless pulsatile mass, although complications such as thrombosis, rupture, or embolism may occur.^1,2)^ Imaging with duplex ultrasound and CT angiography is essential for diagnosis and operative planning. However, current peripheral arterial disease guidelines lack specific recommendations for surgical management of radial artery aneurysms.^4)^ As spontaneous thrombosis or rupture poses a risk, surgical intervention is often warranted. Surgical strategies include ligation and excision, direct arterial repair, or bypass with vein grafts.^2,4–6)^ The decision to revascularize depends on aneurysm location, collateral perfusion, and the completeness of the palmar arch. Preoperative assessment methods such as angiography, magnetic resonance angiography (MRA), and ultrasound are vital.^4,7,8)^ Intraoperative techniques, including digital pulse oximetry under proximal clamping, have also been used to assess distal perfusion and determine the need for reconstruction.^9)^

In our case, preoperative contrast-enhanced CT and ultrasonography confirmed complete occlusion of the ulnar artery. The Allen test further supported the absence of blood flow to the left fingers from sources other than the radial artery. Similar to visceral artery aneurysms, we hypothesized that increased collateral flow through the radial artery territory may have led to aneurysmal formation in distal branches due to flow-related stress. Although increased flow would theoretically result in prominent visualization of the palmar arch, preoperative imaging failed to clearly delineate it. Intraoperatively, retrograde flow from the periphery was confirmed under proximal clamping, and no signs of ischemia, such as pallor or poor capillary refill, were observed. Pulse oximetry readings remained stable during clamping. These findings suggest that contrast-enhanced CT alone may be insufficient for preoperative evaluation and that the palmar arch was likely well-developed in this case. The Allen test is commonly used to assess proximal radial and ulnar artery flow but does not directly reflect the adequacy of distal palmar arch perfusion. Additionally, computed tomography angiography (CTA) may not be sufficient to visualize the distal vasculature, particularly the palmar arch. In our case, the assessment of an underdeveloped palmar arch based solely on ultrasonography, CT imaging, and physical examination may have been inaccurate. More detailed evaluation using catheter angiography or MRA may provide a more accurate assessment of the vascular anatomy surrounding the aneurysm and the development of the palmar arch. However, catheter angiography is invasive and carries risks of complications such as pseudoaneurysm, arterial dissection, and ischemic or necrotic injury. In patients with radial or ulnar artery occlusion, its use must be carefully considered. Noninvasive and potentially capable of providing a more detailed assessment of peripheral blood flow, MRA may have been a more appropriate preoperative modality in this case. Arterial reconstruction was performed due to the absence of objective evidence of sufficient collateral perfusion on preoperative imaging. Had the following conditions been confirmed preoperatively, arterial reconstruction might have been unnecessary, allowing for a less invasive procedure.

- Clear visualization of a well-developed palmar arch on contrast-enhanced CT, MRA, or catheter angiography.
- Preservation of peripheral perfusion independent of the aneurysmal segment, as demonstrated by intraoperative clamping tests or preoperative compression proximal to the aneurysm, with stable pulse oximetry and no ischemic signs.

Although ultrasound or imaging using a hemostatic compression device, such as the TR Band (Terumo, Tokyo, Japan), may be useful, its application to distal hand vasculature is limited due to its proximal placement. Therefore, in our case, intraoperative clamping just proximal to the aneurysm may be the most reliable method for assessing perfusion independent of the aneurysmal segment.

Based on these considerations, we propose the following diagnostic and surgical strategy for similar cases or digital artery aneurysms: Initial evaluation should be performed using CTA. If findings are insufficient, MRA should be added. If both modalities fail to provide adequate information, catheter angiography may be considered, provided there is no significant proximal stenosis or occlusion of the radial or ulnar artery. If a well-developed palmar arch is confirmed and intraoperative clamping or preoperative compression proximal to the aneurysm demonstrates sufficient peripheral perfusion independent of the aneurysmal segment, reconstruction may be omitted. Conversely, if the palmar arch cannot be confidently assessed preoperatively, arterial reconstruction should still be considered, even when satisfactory peripheral perfusion is observed during intraoperative clamping, as demonstrated in the present case. Furthermore, reports exist of cases in which reconstruction was essential in patients with ulnar artery aneurysms and concomitant radial artery occlusion.^10)^ Therefore, in complex vascular pathologies such as this, individualized surgical planning based on accurate preoperative assessment is essential.

## Conclusion

Accurate preoperative assessment of hand arterial anatomy, including the palmar arch, can guide surgical decisions even with ulnar artery occlusion. This case highlights the value of combining preoperative imaging and intraoperative perfusion assessment when considering arterial reconstruction.
